# Electroacupuncture Alleviates LPS-Induced ARDS Through α7 Nicotinic Acetylcholine Receptor-Mediated Inhibition of Ferroptosis

**DOI:** 10.3389/fimmu.2022.832432

**Published:** 2022-02-10

**Authors:** Yiguo Zhang, Li Zheng, Huimin Deng, Di Feng, Song Hu, Lina Zhu, Wenting Xu, Wenyu Zhou, Yu Wang, Keting Min, Qing Zhou, Yuanli Chen, Huanping Zhou, Hao Yang, Xin Lv

**Affiliations:** ^1^ Graduate School, Wannan Medical College, Wuhu, China; ^2^ Department of Anesthesiology, Shanghai Pulmonary Hospital, Tongji University School of Medicine, Shanghai, China

**Keywords:** electroacupuncture, sepsis, ARDS, α7nAchR, ferroptosis

## Abstract

Acute respiratory distress syndrome (ARDS) is an uncontrollable, progressive pulmonary inflammatory disease, and as a common clinical critical disease, there is no effective treatment available. Electroacupuncture (EA) therapy is a type of traditional Chinese medicine physiotherapy that can alleviate the inflammatory response. However, the potential mechanism of EA in the treatment of ARDS is not yet clear. Ferroptosis is a new type of programmed cell death characterized by intracellular iron accumulation and lipid peroxidation. Recently, emerging evidence has shown that ferroptosis is closely related to the occurrence and development of ARDS caused by various pathological factors. Here, we further investigated whether EA-mediated inhibition of ferroptosis in lung tissue could attenuate lipopolysaccharide (LPS)-induced ARDS and explored its underlying mechanisms. In this study, mice were administered LPS intraperitoneally to establish a model of LPS-induced ARDS. We found that EA stimulation could not only reduce the exudation of inflammatory cells and proteins in the alveolar lumen but also significantly alleviate the pathological changes of lung tissue, inhibit the production of proinflammatory cytokines and improve the survival rate of mice. Concurrently, we also found that ferroptosis events occurred in the lung tissue of LPS-induced ARDS mice, manifested by elevated iron levels, ROS production and lipid peroxidation. Intriguingly, our results showed that EA stimulation at the Zusanli (ST36) acupoint activated α7 nicotinic acetylcholine receptor (α7nAchR) in lung tissue mainly through the sciatic nerve and cervical vagus nerve, thus exerting anti-ferroptosis and pulmonary protective effects. Additionally, these effects were eliminated by methyllycaconitine (MLA), a selective antagonist of α7nAchR. *In vitro* experiments, activation of α7nAchR protected alveolar epithelial cells from LPS-induced ferroptosis. Furthermore, our experiments showed that the pulmonary protective effects of EA stimulation were effectively reversed by erastin, a ferroptosis activator. Collectively, we demonstrated that EA stimulation could alleviate LPS-induced ARDS by activating α7nAchR to inhibit LPS-induced ferroptosis in alveolar epithelial cells. Targeting and regulating ferroptosis in alveolar epithelial cells may be a potential intervention approach for the treatment of LPS-induced ALI/ARDS in the future.

## Introduction

Sepsis is a life-threatening organ dysfunction caused by excessive inflammation in response to infection and is associated with high morbidity and mortality. It has been reported that approximately 50% of patients suffering from sepsis will develop acute lung injury (ALI)/acute respiratory distress syndrome (ARDS) (1, 2). Sepsis-induced ALI/ARDS is a clinical syndrome characterized by diffuse damage to alveolar epithelial cells and capillary endothelial cells, resulting in increased alveolar-capillary permeability and lung oedema, which will lead to acute dyspnoea and hypoxemia (3). Although great progress has been made in modern medical research, there is no effective treatment strategy for ALI/ARDS patients, and the mortality rate remains as high as 40% (4, 5).

Acupuncture stimulation is a type of traditional Chinese medicine (TCM) physiotherapy with a history of thousands of years that regulates the physiological conditions of the corresponding internal organs by stimulating specific body parts (acupoints) (6). Electroacupuncture (EA), as a treatment method combining acupuncture and electrophysiological techniques, is commonly used in clinical practice and basic research. Recently, several studies have demonstrated that EA treatment can decrease the production of proinflammatory cytokines, suppress excessive inflammatory responses, and alleviate organ dysfunction. For instance, Liu et al. (7) reported that EA stimulation reduces the release of inflammatory factors through neural regulation, thereby inhibiting the systemic inflammatory response induced by endotoxin; Yang et al. (8) confirmed that EA treatment inhibits the production of proinflammatory factors, attenuates the inflammatory response of the intestine and promotes gastrointestinal peristalsis. Recent studies have also shown that EA stimulation attenuates excessive immune responses in the lung tissue and relieves lung damage by inhibiting the activation of the nod-like receptor pyrin domain-containing 3 (NLRP3) inflammasome and the production of inflammatory exosomes (9, 10). Although these studies have shown that EA treatment exerts an anti-inflammatory impact on ALI/ARDS induced by a variety of inflammatory diseases, the underlying mechanism has not yet been clarified.

The vagus nerve regulates immune function and the production of proinflammatory factors through the cholinergic anti-inflammatory pathway. The α7 nicotinic acetylcholine receptor (α7nAchR), as a potential target of the cholinergic anti-inflammatory pathway, is mainly activated by acetylcholine (Ach) released from the axon terminals of cholinergic neurons to regulate the immune response (8). α7nAchR is mainly expressed on macrophages and other immune cells. Several studies have confirmed that electrical stimulation of the left cervical vagus nerve can reduce sepsis-induced ALI/ARDS by releasing Ach and activating α7nAchR on immune cells (11–14). Interestingly, α7nAchR is also expressed on alveolar epithelial cells, and alveolar epithelial cells are a major cellular target regulated by the cholinergic anti-inflammatory pathway (15, 16). Several studies have confirmed that EA stimulation exerts anti-inflammatory effects mainly by activating α7nAchR in a variety of inflammatory diseases (8, 17–20). In addition, the inflammatory response induced by lipopolysaccharide (LPS) can be reduced by activating α7nAchR on alveolar epithelial cells (21).

Ferroptosis has been recognized as an iron-dependent and different form of cell death from other classical types triggered by lipid peroxidation, characterized by intracellular iron accumulation, production of reactive oxygen species (ROS) and impairment of the antioxidant system, including inactivation of glutathione peroxidase 4 (GPX4) and reduced expression of the light chain subunit SLC7A11 (also known as xCT) of the cystine/glutamate reverse transporter xc-system, which is responsible for the transport of cysteine and provides the raw material for the synthesis of the reductant glutathione (GSH) (22, 23). Erastin induces ferroptosis by specifically inhibiting SLC7A11 and resulting in a decrease in GSH synthesis (24). Fe^2+^ is an essential regulator of normal physiological metabolism, which can be combined with ferritin to form ferritin light chain and ferritin heavy chain 1 (FTH1). In the process of ferroptosis, the expression of FTH1 decreases and excessive iron accumulates in the cell, which promotes the production of ROS through the Fenton reaction, thereby facilitating the occurrence of cellular ferroptosis events (25). More importantly, it plays an important role to maintain the dynamic balance of the oxidation system and antioxidant system in the body in reducing LPS-related ALI/ARDS and improving prognosis (26).

Alveolar epithelial cells are important constituent cells of the alveolar epithelial-endothelial barrier structure and can be involved in the repair of the alveolar barrier structure after damage (27). The alveolar barrier structure is the first line of innate immune defence in the lungs, and alveolar epithelial cells are often the most vulnerable site, causing elevated permeability and diffuse pulmonary oedema, which in turn exacerbates the extent of the damage in the course of acute lung injury. However, in the course of ALI/ARDS caused by various pathological factors, several studies have revealed the relationship between alveolar epithelial cell injury and ferroptosis, and that inhibition of ferroptosis in alveolar epithelial cells can alleviate lung injury. For example, Fan et al. (28) found that melatonin inhibits ferroptosis in epithelial cells and attenuates PM2.5-related lung injury by regulating nuclear factor-erythroid 2-related Factor 2(Nrf2). Xu et al. (26) reported that puerarin can suppress the ferroptosis of lung epithelial cells and alleviate the inflammatory response of sepsis-induced lung injury, and Xu et al. (29) confirmed that ischaemia–reperfusion (IR)-induced lung injury can be improved by inhibiting ferroptosis and acyl-CoA synthetase long-chain family member 4 (ACSL4). A recent study confirmed that EA stimulation can suppress ferroptosis by downregulating oxidative stress and ferroptosis-related proteins (30). It is obvious from the above studies that ferroptosis plays an important role in the progression of lung injury; however, the mechanism underlying the therapeutic effect of EA stimulation on ferroptosis of alveolar epithelial cells in LPS-induced ALI/ARDS is unclear. Thus, the purpose of this study was to investigate whether EA stimulation can exert a pulmonary protective effect by inhibiting ferroptosis through activation of α7nAchR on alveolar epithelial cells.

## Materials and Methods

### Ethics Statement

All experiments and surgical procedures were approved by the Animal Care and Use Committee of the Tongji University School of Medicine, adhered to the recommendations in the Guide for the Care and Use of Laboratory Animals published by the National Institutes of Health.

### Animal

C57BL/6 male mice (6-8weeks, 22-25g) were obtained from Zhejiang Vital River Laboratory Animal Technology Co., Ltd. (Zhejiang, China). All experimental mice were maintained in a standard animal care room with the conditions of 12-hour light and 12-hour dark cycle, given adequate chow and had free access to water. The mice were adapted to the breeding environment for one week before the start of the formal experimental study.

### Electroacupuncture (EA) Intervention

Before the EA stimulation intervention, mice were anaesthetized by inhalation of isoflurane through a small animal gas anesthesia machine and placed on a warming pad to sustain their temperature. EA intervention was applied at the bilateral Zusanli (ST36) acupoints by inserting acupuncture needles at a depth of 3 mm. The acupoint is located approximately 4 mm below the knee joint, 1-2 mm lateral to the anterior tibial tuberosity. Electrical stimulation was performed with the current intensity of 0.5 mA, a frequency of 4/20 Hz and the stimulation time of 20 minutes for 3 consecutive days by using a HANS acupoint nerve stimulator (HANS-200A, Nanjing, China). To investigate the effect of EA stimulation at the ST36 acupoint on LPS-induced lung injury and mortality, EA stimulation was performed 1 hour after intraperitoneal injection of LPS. At the same time, acupuncture needles without electrical stimulation (0mA) were inserted into the same acupoints as a sham EA group (SEA).

### Selective Neurotomies

**Figure 1:**
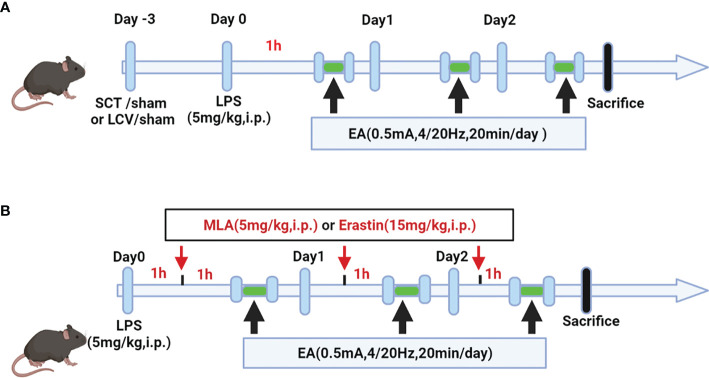
Schematic diagram showing the detail of EA stimulation, surgical approach and drug treatment in LPS-induced ALI/ARDS mouse model. **(A)** Mice were performed with selective neurotomy 3 days prior to LPS (5 mg/kg) administration, after which the mice were then treated with EA stimulation. **(B)** Mice were injected intraperitoneally with LPS (5mg/kg) 1 h prior to MLA (5mg/kg) or erastin (15mg/kg) administration, after which the mice were then treated with EA stimulation.

Selective neurotomies were conducted prior to EA stimulation (**Figure 1A**). Sciatic nerve transection (SCT): The mice were anaesthetized with isoflurane by inhalation through a small animal gas anesthesia machine and the bilateral hindlimbs were shaved and disinfected. The skin incision was performed on the posterior lateral thigh of the mice. The sciatic nerve was obtusely detached, exposed, and a 1 cm long portion of the nerve was transected, as previously depicted by Torres-Rosas et al. (31). For the sham group, the same surgical procedure was made, but without disconnection after the mice were anaesthetized. Left cervical vagotomy (LCV): It was operated as previously depicted by Zhai et al. (32). The mice are anaesthetized and a vertical incision was made along the midline of the neck to isolate the thyroid gland. The carotid artery and the vagal nerve course were observed by obtusely separating the sternocleidomastoid muscle. The left cervical vagus nerve was fixed with a 4.0 surgical suture and excised about 1 cm. For the sham group, mice were anaesthetized and the same surgical procedure was performed, but without disconnection.

### LPS-Induced ALI/ARDS Model and Drugs Treatment

The mice were randomly divided into the various groups (n=4-6 mice per group). To construct LPS-induced ALI/ARDS model in mice, lipopolysaccharide (LPS, Escherichia coli,0111: B4; Sigma-Aldrich, St. Louis, MO, USA) was dissolved in sterile phosphate buffered saline (PBS) and injected intraperitoneally at a dose of 5 mg/kg. Mice in the control group were injected intraperitoneally with equal volume of sterile PBS. For the EA treatment group, EA stimulation was performed 1 h after LPS injection for three consecutive days. Methylaconitine (MLA, 5 mg/kg; Tocris Bioscience), a selective α7nAchR antagonist, was dissolved in sterile saline and injected intraperitoneally 1h before EA treatment (**Figure 1B**). The ferroptosis agonist Erastin (15mg/kg; AdooQ Bioscience, Irvine, CA) was dissolved in 5% dimethyl sulfoxide (DMSO)/corn oil and injected intraperitoneally 1h prior to EA treatment (**Figure 1B**). The selection of dose and time for MLA and Erastin administration were based on the results of previous studies (33, 34) and our pre-experiments. At the selected time point (day 3 after LPS injection), all mice were executed under anesthesia and samples were collected.

### Cell Culture and Intervention

The Mouse lung epithelial cells line MLE-12 cells were obtained from the American Type Culture Collection (ATCC) and grown in DMEM/F12 medium (Gibco, CA, USA) containing 10% fetal bovine serum (Gibco, CA, USA) and 1% penicillin/streptomycin (Gibco, CA, USA). MLE-12 cells were cultured at 37°C incubators in a humidified atmosphere of 5% CO2. We used 10ug/ml LPS to stimulate MLE-12 cells for 24h. Additionally, the MLE-12 cells were treated with various drug interventions, such as vehicle, PNU-282987 (30uM) and Erastin (10uM) for different times, depending on the experimental needs. In this experiment, one sample constituted an independent replicate, and at least three replicates were performed for samples in each group.

### Cell Viability Determination

The CCK-8 assay kit (Dojindo, Kumamoto, Japan) was used to measure the cell viability of MLE-12 cells when they were stimulated with different drugs according to the manufacturer’s instructions. Cells were inoculated into 96-well plates at a density of 5000 cells per well and treated with Erastin (10uM), LPS (10µg/ml), PNU-282987 (30uM) or the same volume of DMSO at various time points when cell growth reached about 60%. After 24 h, 10 ul of working solution was added to each well of the 96-well plate and incubated in a 37°C incubator for 2 h. Next, the absorbance at 450 nm was detected by a microplate reader (Bio-Rad, Hercules, CA).

### Lung Histopathological and Injury Score Analysis

The lung tissues of mice were fixed in 4% paraformaldehyde for at least 24 hours and the samples were then paraffin embedded. Paraffin blocks were sectioned at a thickness of 5 µm and stained with hematoxylin and eosin (H&E). Finally, as described previously (35), the severity of lung injury was scored pathologically in four main aspects: alveolar congestion, pulmonary hemorrhage, infiltration of neutrophils in airspace or vessel wall, and the thickness of alveolar wall/hyaline membrane formation. Each scoring parameter was assessed in turn on a scale of 0 to 4: 0 (minimal injury), 1 (mild injury), 2 (moderate injury), 3 (severe injury), and 4 (maximum injury). The sum of the four parameters represents the final lung injury score.

### Measurement of Protein Concentration and Inflammatory Cell Count in BALF

According to our previous study (35), the bronchoalveolar lavage fuid (BALF) samples were centrifuged at 800×g for 5 minutes. The supernatant was collected and the protein content was determined using the BCA protein assay kit (Thermo Scientific, Rockford, Ill). The cell pellet was resuspended in 100μL PBS and then cells were stained with Wright-Giemsa (Solarbio, Beijing, China) according to the manufacturer’s protocols. The number of inflammatory cells were quantified using a light microscope. Under the microscope, 200 cells/slice were counted at ×40 magnification.

### Lung Tissue Wet/Dry (W/D) Weight Ratio Analysis

After the mice were sacrificed, the right lung tissue was removed, weighed and recorded as wet weight (W). Subsequently, the wet lung tissues were placed in an oven at 80°C for 24 hours and weighed three times at different time points chosen until the weight no longer changed, and then their dry weight (D) was measured. Next, the occurrence of pulmonary oedema was evaluated by calculating the W/D ratio.

### RNA Extraction and Real-Time Quantitative PCR

Total RNA was isolated from MLE-12 cells or the lung tissues using Trizol reagent (Invitrogen, Calif). After quantifying the concentration and purity of RNA, the gDNA was removed and complementary DNA synthesis was conducted using Prime Script RT Master Mix (Takara, China). Subsequently, real-time qPCR was conducted on a Light Cycler 480 real-time PCR system (Roche, Rotkreuz, Switzerland) using iTaq universal SYBR Green Super mix (Bio-Rad, Hercules, Calif). The relative expression levels of the target genes were standardized to β-actin and calculated by using the 2^-ΔΔCT^ method. The primer sequences are shown in **Supplementary Table 1**.

### Western Blot Analysis

Total protein of MLE-12 cells or the lung tissues were lysed and extracted using RIPA lysis buffer containing a protease inhibitor, a phosphatase inhibitor, and Phenylmethanesulfonyl fluoride (Beyotime, Shanghai, China). Protein concentrations were quantified using the BCA protein assay kit (Thermo Scientific, Rockford, Ill). The target proteins were separated by 12% SDS-PAGE and transferred to PVDF membranes (Millipore, Billerica, Mass). Furthermore, the PVDF membranes were blocked with 5% Bovine Serum Albumin (BSA) for 1h at room temperature and incubated overnight at 4°C with primary antibodies against α7nAchR (1:1000, Abcam), GPX4(1:1000, Abcam), SLC7A11(1:1000, ABclonal, Wuhan, China), FTH1(1:1000, Cell Signaling Technology), β-actin (1:1000, Cell Signaling Technology). The membranes were washed 3 times with TBST and then incubated with secondary antibody (1:5000, anti-rabbit IgG, Cell Signaling Technology) for 1h. The target proteins were measured using the enhanced chemiluminescence (Thermo Scientific, USA) and western blotting assay system (Bio-Rad, USA). In addition, the relative protein expression was calculated by using Quantity One software (Bio-Rad, USA).

### Detection of ROS

Generation of ROS was detected with ROS Assay Kit (Beyotime, China). In brief, DCFH-DA was diluted to a concentration of 10 uM. MLE-12 cells were washed with PBS firstly and incubated with DCFH-DA at 37°C for 20 min. After that, cells were washed with PBS for 3 times and detected by fluorescent microscopy. *In vivo* experiment, fresh lung tissues were stained with DHE for ROS detection, which was performed as described previously (36). The level of ROS in lung tissues (red fluorescence) was determined by a fluorescence microscope (Olympus, Tokyo, Japan).

### Measurement of Ferroptosis-Related Markers

Levels of Iron, the lipid peroxidation metabolite malondialdehyde (MDA) and the reductant glutathione (GSH) were measured in the lung tissues and MLE-12 cells by using the Iron assay kit (Sigma-Aldrich), the MDA assay kit (Sigma-Aldrich), and the GSH assay kit (Sigma-Aldrich), respectively, in accordance with the manufacturer’s instructions.

### Statistical Analysis

All results in this study were presented as the mean ± standard deviation (SD). Statistical analyses were performed using SPSS 17.0 software (SPSS, Chicago, Ill) and GraphPad Prism 8.0 software. To compare the differences between the two groups, Student’s t-test was employed. However, differences between multiple groups were compared by using a one-way ANOVA analysis and Tukey’s *post hoc* test. These statistical differences were regarded as significant at the level of *p*<0.05.

## Results

### Ferroptosis Is Activated in an LPS-Induced ALI/ARDS Model

**Figure 2:**
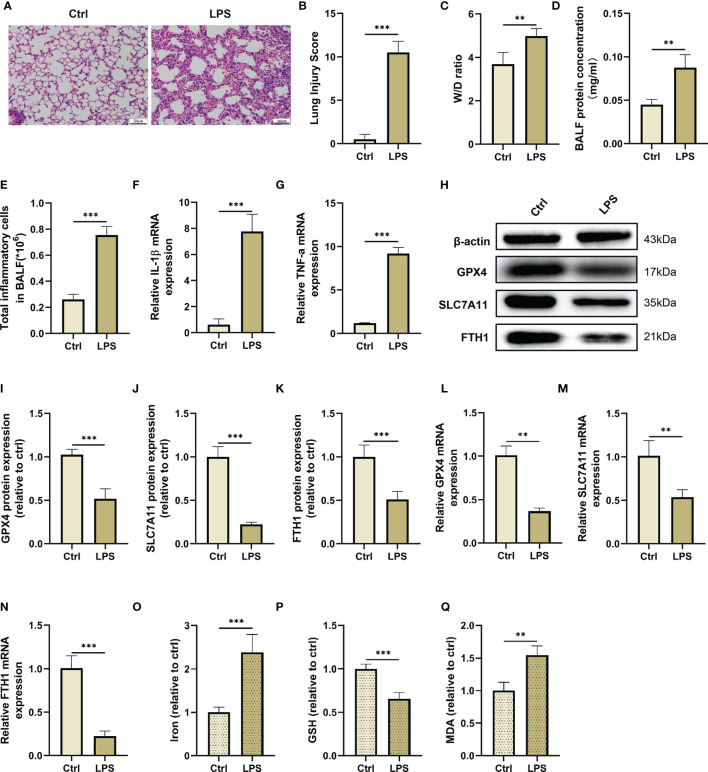
LPS administration induces lung injury and ferroptosis *in vivo*. **(A)** The representative H&E staining of lung tissue sections (scale bar, 50 μm). **(B)** The lung injury score analysis (n = 4). **(C)** The wet/dry ratio of lung tissues (n = 4). **(D)** The protein concentration in BALF (n = 4). **(E)** The number of inflammatory cells in BALF (n = 4). The IL-1β **(F)** and TNF-α **(G)** mRNA levels in lung tissues were detected by real-time qPCR (n = 4). **(H)** The GPX4, SLC7A11 and FTH1 protein levels in lung tissues were detected by western blotting. The relative intensities of GPX4 **(I)**, SLC7A11 **(J)** and FTH1**(K)** (n = 4). The mRNA expression levels of GPX4 **(L)**, SLC7A11 **(M)** and FTH1 **(N)** in the lung tissues were detected by real-time qPCR (n = 4). The contents of Iron **(O)**, GSH **(P)** and MDA **(Q)** in lung tissues (n = 4). Data are expressed as mean ± SD. **p < 0.01 and ***p < 0.001 indicate significant differences from each group.

In this study, we established an LPS-induced ALI/ARDS mouse model by intraperitoneal injection of LPS. First, we observed the survival rate of mice injected intraperitoneally with LPS and found that the mice had the highest mortality rate in the first 3 days (**Supplementary Figure 1C**). We then evaluated pathological changes and acute inflammatory responses in the lung tissues on Day 1, 2, 3 and 4 after LPS administration. The H&E staining and lung injury score results showed that intraperitoneal LPS injection induced significant pathological changes, including incomplete alveolar walls, thickened alveolar septa, diffuse interstitial oedema and inflammatory cell infiltration in the lung tissues in a time-dependent manner, and reached their peak in the first 3 days after injection (**Supplementary Figures 1A, B, D, E**). Similarly, the mRNA expression of proinflammatory cytokines, such as interleukin (IL)-1β and tumor necrosis factor (TNF)-α, in the lung tissues evidently increased in the first 3 days and significantly decreased on Day 4 after LPS injection (**Supplementary Figures 1F, G**). Therefore, a 3-day time point was selected as the interventional time point to be used in the following experiments (**Figures 2A–G**).

Next, we detected makers of ferroptosis, such as iron accumulation, reductant GSH levels, MDA content and lipid peroxidation levels in lung tissues after LPS administration. As shown in this study, the GSH level in the lung tissues was reduced after LPS injection, while intraperitoneal injection of LPS dramatically increased the contents of malondialdehyde (MDA), a final product of lipid peroxidation, and iron in the lung tissues (**Figures 2O–Q**). Furthermore, we also found that LPS administration reduced the mRNA and protein levels of ferroptosis negative regulators, such as GPX4, SLC7A11, and FTH1 in the lung tissue compared with the control group (**Figures 2H–N**). These results suggested that ferroptosis events occured in an LPS-induced ALI/ARDS mouse model.

### Electroacupuncture Inhibites Ferroptosis in Lung Tissues and Alleviates LPS-Induced ALI/ARDS

**Figure 3:**
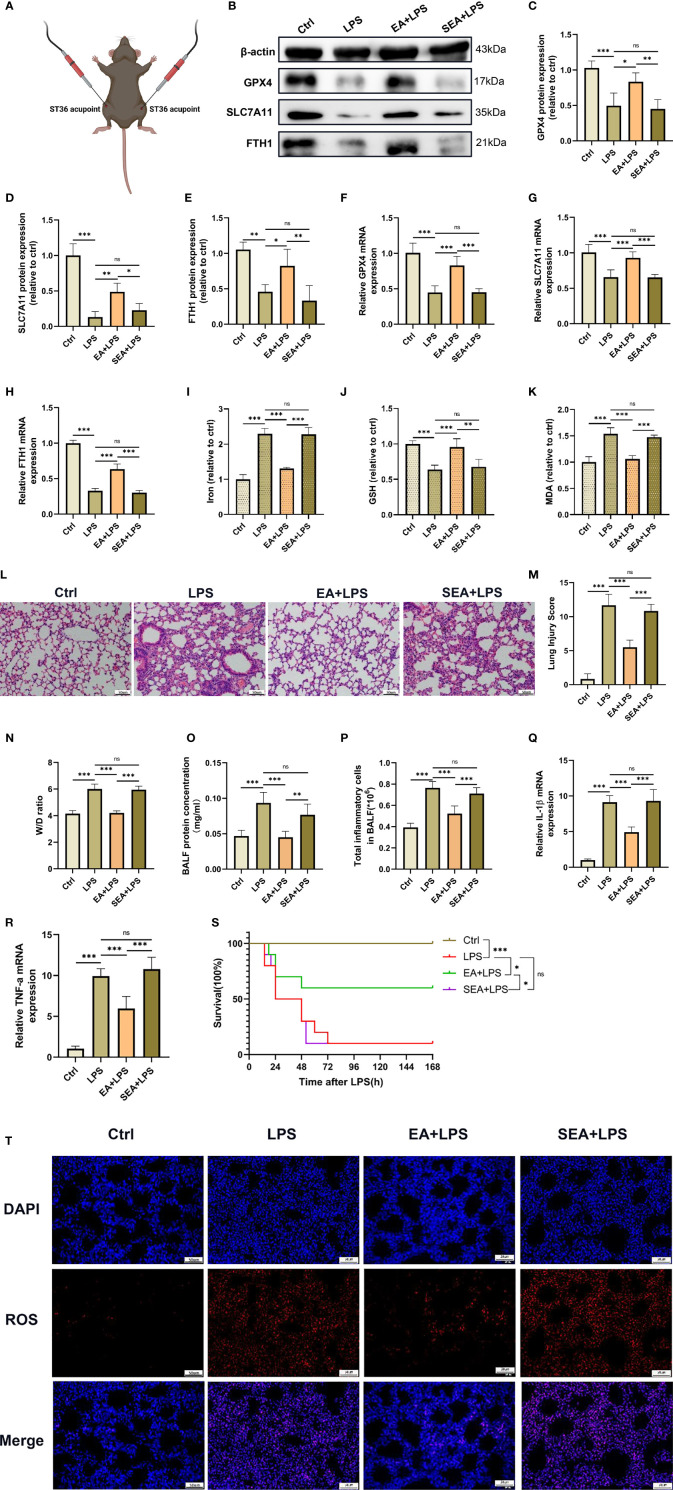
Inhibition of ferroptosis by EA stimulation alleviates LPS-induced ARDS. The mice were injected intraperitoneally with LPS (5mg/kg) for 1 hour followed by EA stimulation at the bilateral Zusanli (ST36) acupoints **(A)**, 20min/day, three days and then the lung tissues were harvested. **(B–H)** The relative mRNA and protein levels of GPX4, SLC7A11, and FTH1 were determined by real-time qPCR and western blotting, respectively (n = 4). The contents of Iron **(I)**, GSH **(J)** and MDA **(K)** in lung tissues (n = 4). **(L)** The representative H&E staining of lung tissue sections (scale bar, 50 μm). **(M)** The lung injury score analysis (n = 4). **(N)** The wet/dry ratio of lung tissues (n = 4). **(O)** The protein concentration in BALF (n = 4). **(P)** The number of inflammatory cells in BALF (n = 4). The IL-1β **(Q)** and TNF-α **(R)** mRNA levels in lung tissues were determined by real-time qPCR (n = 4). **(S)** The survival rate of mice (n =10). **(T)** The ROS level in lung tissues were evaluated by DHE staining (scale bar, 50 μm). Data are expressed as mean ± SD. *p < 0.05, **p < 0.01 and ***p < 0.001 indicate significant differences from each group. ns, no significance.

We next sought to assess the therapeutic effect of EA on LPS-induced ferroptosis and ALI/ARDS in the lung tissues (**Figure 3A**). The results showed that the downregulated GPX4, SLC7A11 and FTH1 mRNA and protein levels in the lung tissues of LPS-challenged mice were all increased after EA treatment; however, sham EA treatment had no significant effect on GPX4, SLC7A11 and FTH1 mRNA and protein levels in the lung tissues of LPS-challenged mice (**Figures 3B–H**). Moreover, as shown in the results, EA treatment remarkably mitigated the LPS-induced upregulation of iron and MDA contents and increased the level of GSH in the lung tissues of LPS-treated mice but not sham EA treatment (**Figures 3I–K**). We also found that after LPS injection, the production of ROS in the lung tissues was increased, while EA treatment markedly inhibited LPS-induced ROS accumulation in the lung tissues (**Figure 3T**). The H&E staining results clearly showed that after treatment with LPS, acute inflammatory responses, such as interstitial oedema, pulmonary architecture destruction and inflammatory cell infiltration, were observed in the lung tissues; however, the aforementioned pathological changes in the lung were evidently attenuated following EA intervention but not by sham EA (**Figure 3L**). In addition, the lung injury score and W/D ratio were also significantly decreased after EA treatment (**Figures 3M, N**). By analysing the BALF results, we found that EA intervention significantly reduced the total protein concentration in BALF and reduced inflammatory cell exudation in mice injected intraperitoneally with LPS but not sham EA treatment (**Figures 3O, P**). We next examined the gene expression of proinflammatory cytokines in lung tissues by real-time qPCR and found that both IL-1β and TNF-α mRNA expression was significantly upregulated in the lung tissues of LPS-treated mice and that EA treatment effectively reduced the gene expression of these two proinflammatory cytokines, whereas sham EA treatment did not produce a significant anti-inflammatory effect (**Figures 3Q, R**). We also verified the role of EA in improving the survival of LPS-induced ALI/ARDS mice. The results showed that EA could improve the survival rate of mice injected intraperitoneally with LPS (**Figure 3S**). These data confirmed that EA stimulation at the ST36 acupoint could effectively inhibit ferroptosis in lung tissues, alleviated the pulmonary inflammation response and improved the survival rate in LPS-induced ALI/ARDS mice.

### EA Suppresses Ferroptosis in Lung Tissues and Alleviates LPS-Induced ALI/ARDS by Activating α7nAchR

**Figure 4:**
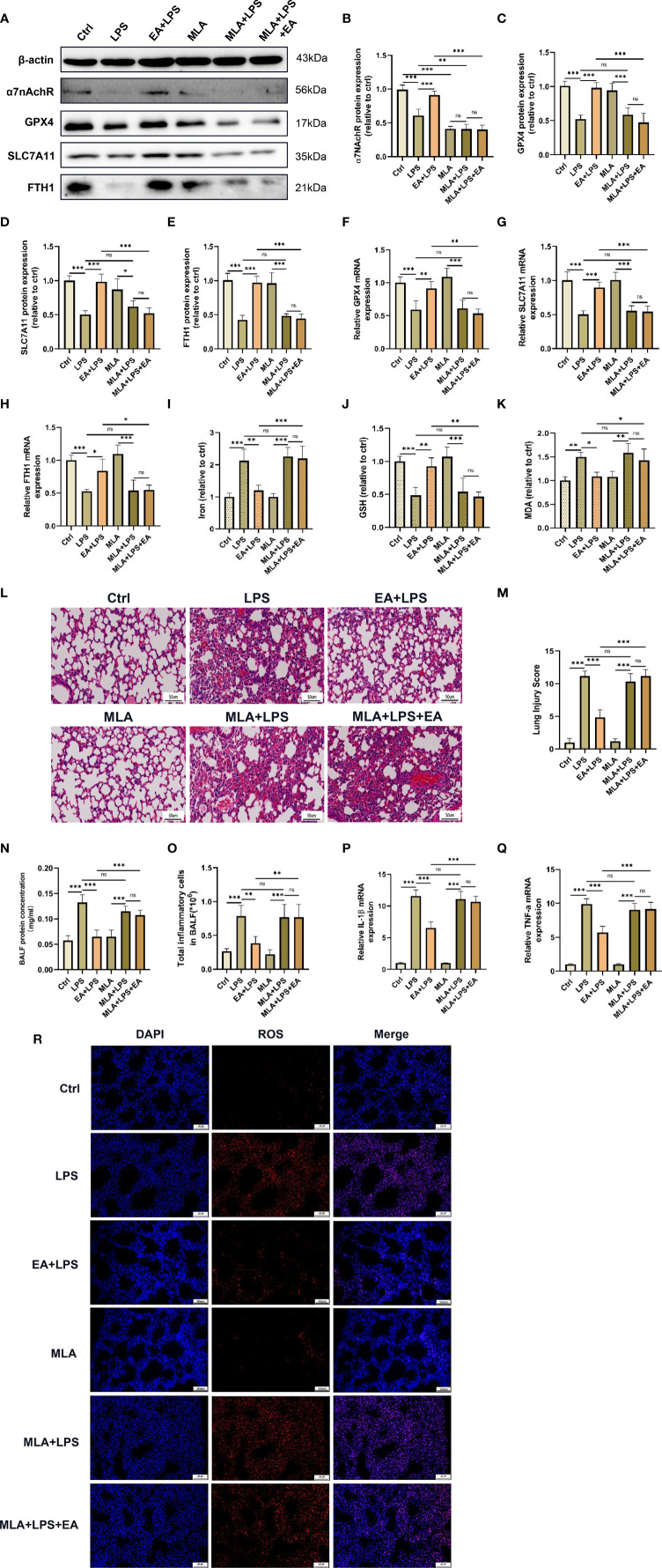
EA suppresses ferroptosis in lung tissue and alleviates LPS-induced ALI/ARDS by activating α7nAchR. The mice were injected intraperitoneally with MLA (5mg/kg) after LPS (5mg/kg) administration for 1 hour followed by EA stimulation, 20min/day, three days. **(A–H)** The relative mRNA and protein levels of α7nAchR, GPX4, SLC7A11, and FTH1 in lung tissues were examined by real-time qPCR and western blotting, respectively (n = 4). The contents of Iron **(I)**, GSH **(J)** and MDA **(K)** in lung tissues (n = 4). **(L)** The representative H&E staining of lung tissue sections (scale bar, 50 μm). **(M)** The lung injury score analysis (n = 4). **(N)** The protein concentration in BALF (n = 4). **(O)** The number of inflammatory cells in BALF (n = 4). The mRNA expression of IL-1β **(P)** and TNF-α **(Q)** in lung tissues (n = 4). **(R)** The ROS level in lung tissues were evaluated by DHE staining (scale bar, 50 μm). Data are expressed as mean ± SD. *p < 0.05, **p < 0.01 and ***p < 0.001 indicate significant differences from each group. ns, no significance.

Previous studies have demonstrated that α7nAchR played an important role in vagal-mediated cholinergic anti-inflammatory pathways, α7nAchR activation was involved in the protective effect of EA stimulation in ALI/ARDS (19, 37). We next investigated whether EA could alleviate LPS-induced ferroptosis in lung tissues by activating α7nAchR (38). The western blot results showed that EA could upregulate the protein expression of α7nAchR in the lung tissues of LPS-treated mice, while the EA-induced upregulation of the expression of α7nAchR protein was significantly inhibited by methyllycaconitine (MLA), a selective α7nAchR antagonist (**Figures 4A, B**). Concurrently, MLA also dampened the upregulation of GPX4, SLC7A11 and FTH1 expression by EA in the lung tissues of LPS-treated mice (**Figures 4A, C–H**). As shown in the results, in the LPS-induced ALI/ARDS model, EA treatment significantly reduced the levels of iron, MDA and ROS in the lung tissues and increased the content of GSH, but these regulatory effects were effectively suppressed by MLA (**Figures 4I–K, R**). H&E staining further revealed that EA treatment markedly inhibited inflammatory cell infiltration, alleviated lung injury, and reduced lung injury scores in LPS-injected mice, while MLA eliminated this protective effect of EA (**Figures 4L, M**). MLA also inhibited the therapeutic effect of EA on the exudation of proteins and inflammatory cells in the BALF of LPS-injected mice (**Figures 4N, O**). Pretreatment with MLA weakened the suppressive effect of EA on the gene expression of IL-1β and TNF-α in lung tissues of LPS-injected mice (**Figures 4P, Q**). These results confirmed that EA treatment inhibited LPS-induced ferroptosis in lung tissues by activating α7nAchR.

### The Pulmonary Protective Effect of EA Is Eliminated by Sciatic Nerve Transection

**Figure 5:**
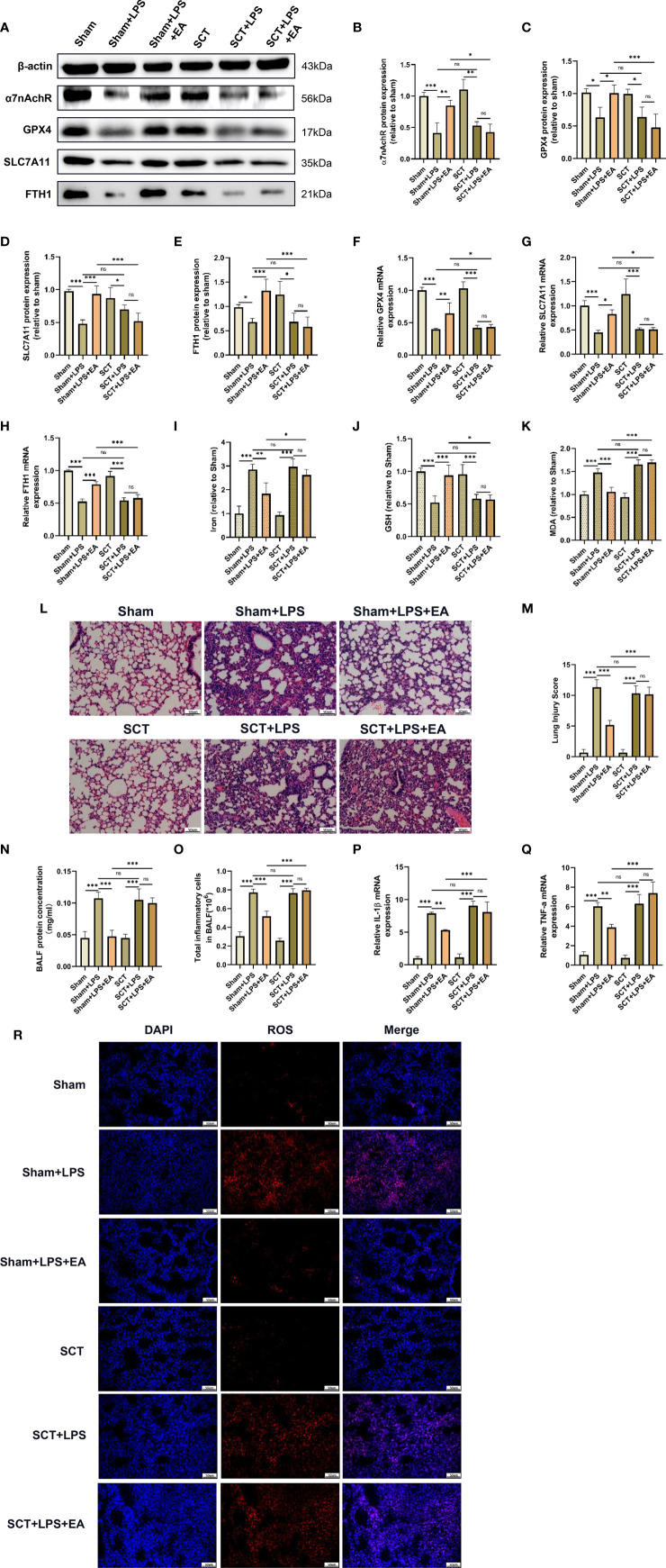
The pulmonary protective effect of EA is eliminated by sciatic nerve transection. Mice were performed with sciatic nerve transection 3 days prior to LPS (5 mg/kg) administration, after which the mice were then treated with EA stimulation, 20min/day, three days. **(A–H)** The relative mRNA and protein levels of α7nAchR, GPX4, SLC7A11, and FTH1 in lung tissues were examined by real-time qPCR and western blotting (n = 4). The contents of Iron **(I)**, GSH **(J)** and MDA **(K)** in lung tissues (n = 4). **(L)** H&E staining of lung tissue sections (scale bar, 50 μm). **(M)** The lung injury score analysis (n = 4). **(N)** The protein concentration in BALF (n = 4). **(O)** The number of inflammatory cells in BALF (n = 4). The mRNA expression of IL-1β **(P)** and TNF-α **(Q)** in lung tissues were examined by real-time qPCR (n = 4). **(R)** The ROS level in lung tissues were evaluated by DHE staining (scale bar, 50 µm). Data are expressed as mean ± SD.*p < 0.05, **p < 0.01 and ***p < 0.001 indicate significant differences from each group. ns, no significance.

The ST36 acupoint is located near the branches of the sciatic nerve, such as the common peroneal nerve and tibial nerve. To explore whether the sciatic nerve is essential for the antiferroptotic effect of EA in lung tissues, we performed sciatic nerve transection. Compared with the EA group, the EA-induced elevation of GPX4, SLC7A11 and FTH1 protein and mRNA expression in the lung tissues of LPS-injected mice was significantly inhibited by bilateral sciatic nerve transection (**Figures 5A, C–H**). We also found that EA stimulation failed to upregulate α7nAchR protein expression in the lung tissues of LPS-injected mice after bilateral sciatic nerve transection (**Figures 5A, B**). As shown in the results, both the reduction in iron and MDA levels and the upregulation of GSH content in the lung tissues of LPS-injected mice evoked by EA stimulation were significantly affected by bilateral sciatic nerve transection (**Figures 5I–K**). In addition, after bilateral sciatic nerve transection, the inhibitory effect of EA on the" production of ROS in lung tissues after LPS stimulation was almost eliminated (**Figure 5R**). We also found no improvement in the symptoms of lung injury and no reduction in lung injury scores in LPS-injected mice after bilateral sciatic nerve transection (**Figures 5L, M**). The results of the BALF analysis further suggested that EA-induced inhibition of protein exudation and inflammatory cell infiltration in the lung tissues of LPS-injected mice was evidently eliminated by bilateral sciatic nerve transection (**Figures 5N, O**). As shown in this study, real-time qPCR detection revealed that EA treatment failed to suppress the gene expression levels of the inflammatory factors IL-1β and TNF-α in LPS-stimulated lung tissues after bilateral sciatic nerve transection (**Figures 5P, Q**). The above evidence indicated that the sciatic nerve was essential for the antiferroptotic effect of ST36 acupoint EA stimulation in LPS-induced ALI/ARDS.

### The Vagus Nerve Is Involved in the Pulmonary Protective and Antiferroptotic Effects of EA in LPS-Induced ALI/ARDS

**Figure 6:**
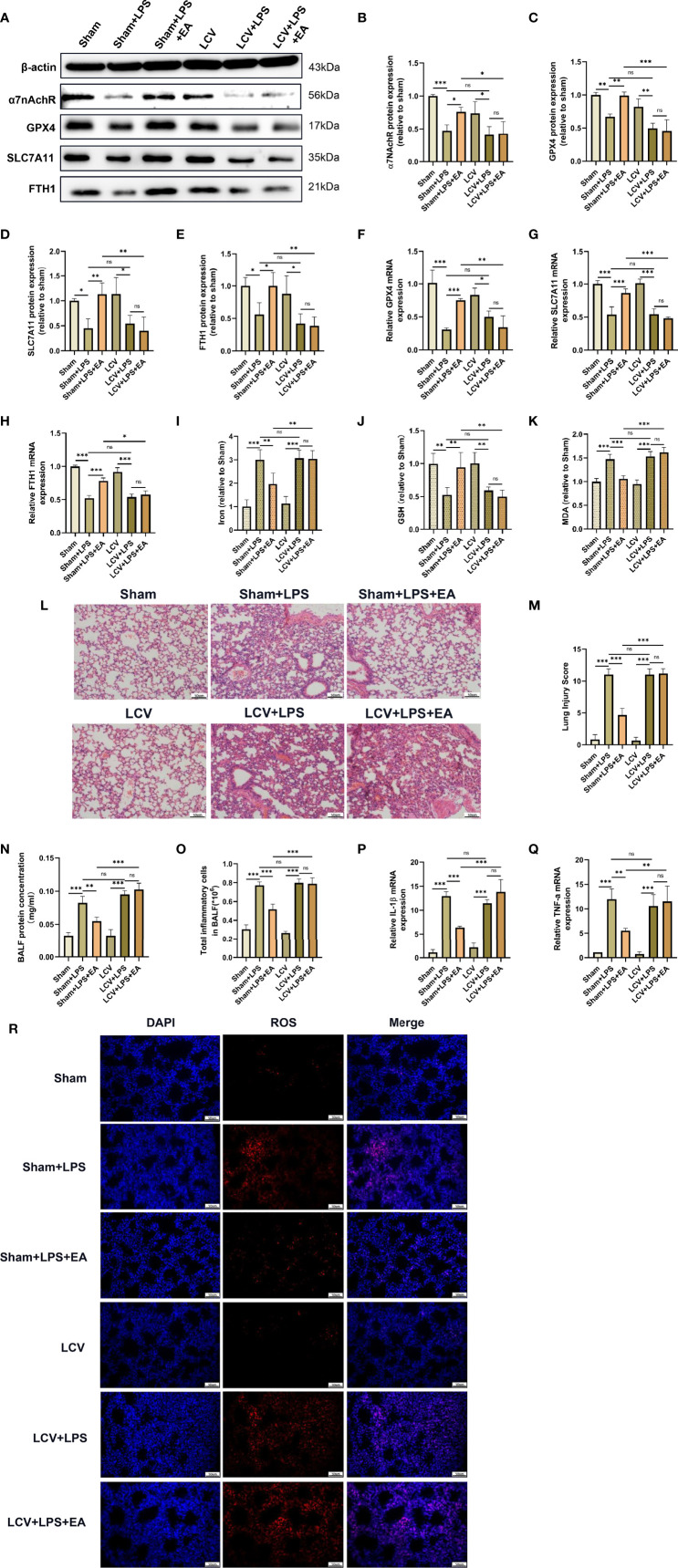
The pulmonary protective and antiferroptotic effects of EA were eliminated by left cervical vagotomy in LPS-induced ALI/ARDS. Mice were performed with cervical vagotomy 3 days prior to LPS (5 mg/kg) administration, after which the mice were then treated with EA stimulation, 20min/day, three days. **(A–E)** The relative protein levels of α7nAchR, GPX4, SLC7A11, and FTH1 in lung tissues were examined by western blotting (n = 4). The mRNA expression of GPX4 **(F)**, SLC7A11 **(G)** and FTH1 **(H)** in lung tissues were examined by real-time qPCR (n = 4). The contents of Iron **(I)**, GSH **(J)** and MDA **(K)** in lung tissues (n = 4). **(L)** H&E staining of lung tissue sections (scale bar, 50 μm). **(M)** The lung injury score analysis (n = 4). **(N)** The protein concentration in BALF (n = 4). **(O)** The number of inflammatory cells in BALF (n = 4). The IL-1β **(P)** and TNF-α **(Q)** mRNA levels in lung tissues were examined by real-time qPCR (n = 4). **(R)** The ROS level in lung tissues were evaluated by DHE staining (scale bar, 50 μm). Data are expressed as mean ± SD. *p < 0.05, **p < 0.01 and ***p < 0.001 indicate significant differences from each group. ns, no significance.

Numerous studies have shown that the vagal nerve-mediated cholinergic anti-inflammatory pathway is responsible for regulating immune functions and the inflammatory response, and EA stimulation at the sciatic nerve, such as the ST36 acupoint, controls systemic inflammation and attenuates organ dysfunction by inducing vagal activation (7, 19, 31, 39). To explore whether the vagal nerve is involved in the protective effect of EA on LPS-induced ferroptosis in mice, we performed a left cervical vagotomy before intraperitoneal injection of LPS (8). Compared with the EA group, we found that the expression of α7nAchR protein was not elevated in the lung tissues of LPS-injected mice with left cervical vagotomy after EA treatment (**Figures 6A, B**). In addition, the effect of EA on the protein and mRNA expression of GPX4, SLC7A11 and FTH1 in the lung tissues of LPS-injected mice was significantly inhibited after left cervical vagotomy (**Figures 6A, C–H**). As shown in the results, both the reduction in iron and MDA levels and upregulation of GSH content in the lung tissues of LPS-injected mice evoked by EA stimulation were suppressed by left cervical vagotomy (**Figures 6I–K**). Consistently, the inhibitory effect of EA on LPS-induced ROS production in lung tissues was almost eliminated by left cervical vagotomy (**Figure 6R**). Next, H&E staining, lung injury scores and BALF analysis showed that left cervical vagotomy abolished the effects of EA in alleviating the pulmonary inflammatory response, decreasing lung injury scores, and suppressing protein exudation and inflammatory cell aggregation (**Figures 6L–O**). As shown in this study, after left cervical vagotomy, EA treatment failed to inhibit the gene expression of the proinflammatory factors IL-1β and TNF-α in the lung tissues of LPS-injected mice (**Figures 6P, Q**). The above results suggested that EA stimulation at the ST36 acupoint effectively inhibited LPS-induced ferroptosis and reduced the inflammatory response in lung tissues *via* activation of the vagal nerve.

### EA Treatment Attenuates LPS-Induced ALI/ARDS by Inhibiting Ferroptosis

**Figure 7:**
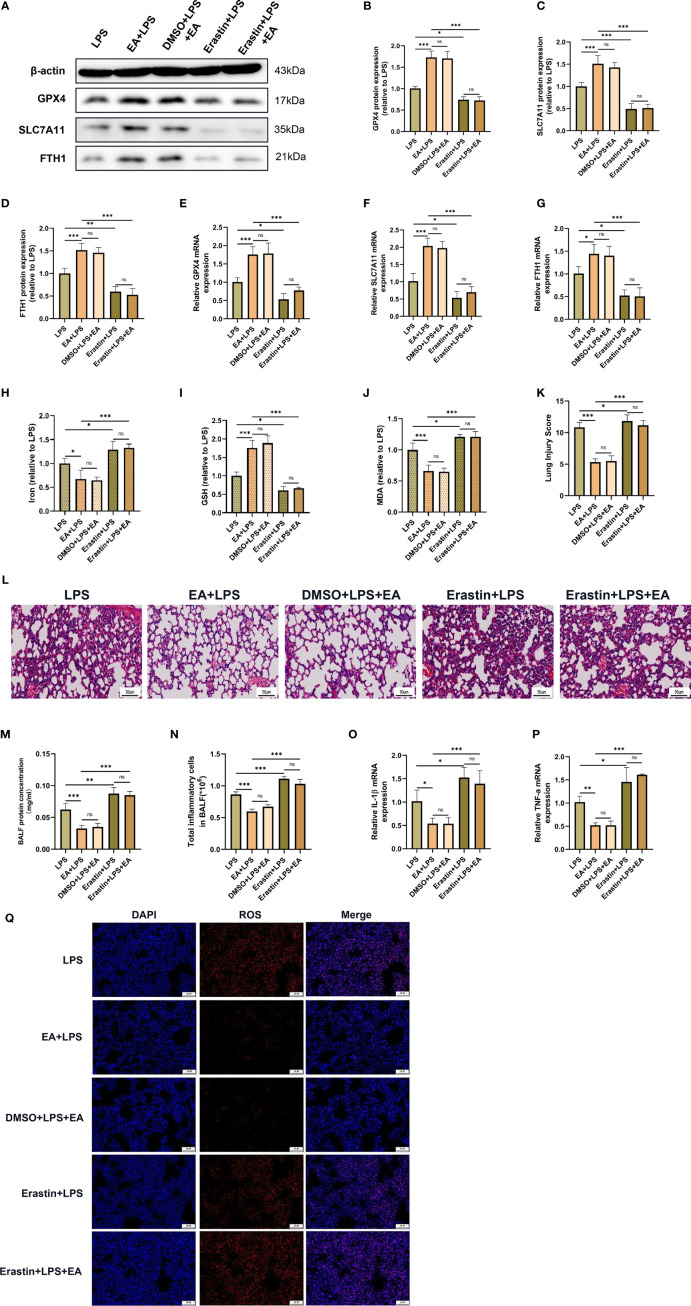
EA stimulation alleviates LPS-induced ALI/ARDS by inhibiting ferroptosis. Mice were injected intraperitoneally with LPS (5mg/kg) 1 h prior to erastin (15mg/kg) administration, after which the mice were then treated with EA stimulation, 20min/day, three days. **(A–G)** The relative mRNA and protein levels of GPX4, SLC7A11 and FTH1 in lung tissues were examined by real-time qPCR and western blotting (n = 4). The contents of Iron **(H)**, GSH **(I)** and MDA **(J)** in lung tissues (n = 4). **(K)** The lung injury score analysis (n = 4). **(L)** H&E staining of lung tissue sections (scale bar, 50 µm). **(M)** The protein concentration in BALF (n = 4). **(N)** The number of inflammatory cells in BALF (n = 4). The IL-1β **(O)** and TNF-α **(P)** mRNA levels in lung tissues were examined by real-time qPCR (n = 4). **(Q)** The ROS level in lung tissues were evaluated by DHE staining (scale bar, 50 μm). Data are expressed as mean ± SD. *p < 0.05, **p < 0.01 and ***p < 0.001 indicate significant differences from each group. ns, no significance.

We further investigated whether EA treatment mitigated LPS-induced ALI/ARDS by attenuating ferroptosis. Erastin, an inducer of ferroptosis, was applied in this study. As shown in the figures, erastin significantly hampered EA treatment-induced upregulation of GPX4, SLC7A11 and FTH1 expression and GSH levels and reversed EA treatment-induced downregulation of the levels of MDA, iron and ROS in the lung tissues of LPS-injected mice (**Figures 7A–J, Q**). Erastin also effectively abolished the protective effect of EA on LPS-induced lung injury, with the main pathological features being widened alveolar septa, haemorrhage in lung tissues and massive inflammatory cell infiltration (**Figures 7K, L**). The results of BALF analysis indicated that after treatment with erastin, EA intervention failed to reduce the protein content and the total number of inflammatory cells in BALF (**Figures 7M, N**). We further detected the gene expression of IL-1β and TNF-α in lung tissues and found that erastin treatment reversed the inhibitory effect of EA on LPS-induced ALI/ARDS and the upregulation of inflammatory factor expression (**Figures 7O, P**). These results suggested that EA treatment mitigated LPS-induced ALI/ARDS by attenuating ferroptosis.

### Activating α7nAchR Protects Against LPS-Induced Damage and Ferroptosis in MLE-12 Cells

**Figure 8:**
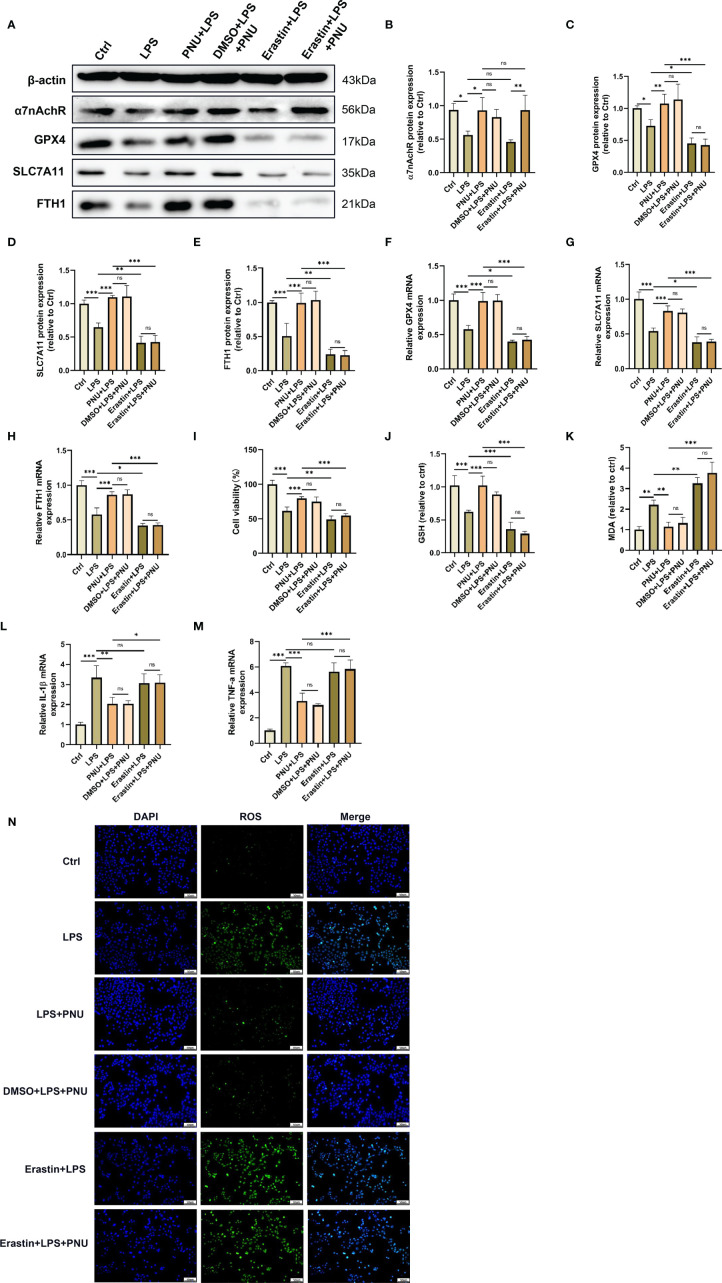
Activating α7nAchR protects against LPS-induced damage and ferroptosis in MLE-12 cells. MLE-12 cells were treated with erastin (10 uM) 3 h prior to LPS (10 ug/ml) treatment, after which the MLE-12 cells were then treated with PNU-282987 (30 uM). **(A)** The protein expression levels of α7nAchR **(B)**, GPX4 **(C)**, SLC7A11 **(D)** and FTH1 **(E)** in MLE-12 cells were examined by westing blotting. The mRNA expression levels of GPX4 **(F)**, SLC7A11 **(G)**, FTH1 **(H)**, IL-1β **(L)** and TNF-α **(M)** in MLE-12 cells were examined by real-time qPCR (n = 6). **(I)** Cell viability was detected by an CCK8 assay (n = 6). The cellular contents of GSH **(J)** and MDA **(K)** (n = 6). **(N)** The cellular ROS level in MLE-12 cells were evaluated by ROS Assay Kit (scale bar, 50 μm). Data are expressed as mean ± SD. *p < 0.05, **p < 0.01 and ***p < 0.001 indicate significant differences from each group. ns, no significance.

Alveolar epithelial cells are the essential component of the alveolar epithelial-endothelial barrier and may be firstly affected by ARDS. Excessive damage to alveolar epithelial cells could lead to an increase in alveolar-capillary permeability and cause pulmonary oedema (40–42). In addition, several studies have shown that ferroptosis occurs in alveolar epithelial cells following LPS stimulation (26, 43). To further investigate the role of the α7nAchR-mediated signalling pathway in LPS-induced ferroptosis in alveolar epithelial cells, the highly selective α7nAchR agonist PNU-282987 and the ferroptosis inducer erastin were used *in vitro*. As expected, PNU-282987 significantly elevated the protein expression of α7nAchR in LPS-treated mouse alveolar epithelial cells (MLE-12) (**Figures 8A, B**). The results in the figure revealed that PNU-282987 significantly inhibited LPS-induced MLE-12 cell injury, manifested by the increased expression of GPX4, SLC7A11 and FTH1 and the increased levels of cell viability and GSH (**Figures 8A, C–J**). The contents of MDA and ROS in LPS-treated MLE-12 cells were also decreased by PNU-282987 (**Figures 8K, N**). Similarly, after pretreatment with erastin, the antiferroptotic effect of PNU-282987 was eliminated in LPS-treated MLE-12 cells. In addition, the inhibitory effect of PNU-282987 on the LPS-induced inflammatory response in MLE-12 cells was also reversed by erastin, as evidenced by a significant increase in IL-1β and TNF-α levels (**Figures 8L, M**). These results suggested that activation of α7nAchR relieved the inflammatory response and protected alveolar epithelial cells from LPS-induced ferroptosis.

## Discussion

In the present study, we explored the pulmonary protective effect of EA treatment and its potential mechanism in LPS-induced ferroptosis of alveolar epithelial cells. We revealed for the first time that EA stimulation of the ST36 acupoint could significantly attenuate LPS-induced lung injury by inhibiting ferroptosis in lung tissues through activation of α7nAchR. More importantly, the inhibitory effects of EA on both the inflammatory response and ferroptosis in LPS-induced ALI/ARDS were dependent on the sciatic nerve and cervical vagus nerve. Moreover, our *in vitro* experiments also showed that activation of α7nAchR attenuated the inflammatory response and protected LPS-treated lung epithelial cells from ferroptosis. Collectively, the present study suggested that EA stimulation at the ST36 acupoint inhibited ferroptosis in alveolar epithelial cells by activating α7nAchR *via* the sciatic nerve and cervical vagus nerve, and might be a potential mechanism by which EA treatment alleviates LPS-induced ALI/ARDS.

α7nAchR is a target of a cholinergic anti-inflammatory signalling pathway that is widely expressed on the surface of alveolar epithelial and immune cells and is activated primarily by the release of acetylcholine from vagal efferent nerves (44). Previous studies have demonstrated that EA treatment can exhibit potent organ protective effects in various disease conditions, such as postoperative ileus (8), doxorubicin-induced cardiotoxicity (45), cerebral IR injury (46), and hepatic IR injury (47), and this protective role of EA stimulation is mostly dependent on the activation of α7nAchR. It is worth noting that the therapeutic effects of EA therapy on a variety of organs are directly related to multiple signalling pathways mediated by α7nAchR, in which the α7nAchR-mediated Janus kinase 2 (JAK2)/signal transducer and activator of transcription 3(STAT3) signalling pathway (48), high-mobility group Box 1(HMGB1)/nuclear factor-κB (NF-κB) signalling pathway (49), and NLRP3 signalling pathway (18) have been regarded as significantly associated with the organ-protective effects of EA treatment. In the present study, we found that EA could upregulate the expression of α7nAchR to inhibit the production of proinflammatory factors, reduce protein and inflammatory cell exudation in the alveolar lumen, and improve histopathological changes in lung tissues, thereby alleviating LPS-induced ALI/ARDS. However, the pulmonary protective effect of EA stimulation was reversed with the application of the α7nAchR-specific antagonist MLA. In addition, *in vitro* experiments, we found that activation of α7nAchR on alveolar epithelial cells reduced the production of proinflammatory factors and enhanced cell viability.

Iron is an essential element for maintaining normal physiological conditions, including haematopoiesis, synthesis of many enzymes, immune regulation and energy metabolism. However, intracellular iron accumulation also has toxic effects in terms of biological function, generating large amounts of oxygen free radicals, which in turn cause damage to intracellular DNA, proteins, and cell organelles (50). Ferroptosis is a nonapoptotic form of regulated cell death that is characterized by intracellular iron overload and lipid peroxidation product accumulation. Notably, ferroptosis has been shown to occur in the lung tissues of LPS-stimulated mice and alveolar epithelial cells (26). In this study, we demonstrated that ferroptosis was significantly activated in LPS-induced ALI/ARDS mice with elevated Fe^2+^ and MDA levels, reduced levels of the reducing agent GSH, and downregulated gene and protein expression of GPX4, SLC7A11 and FTH1. Recent studies have further confirmed that inhibition of ferroptosis in alveolar epithelial cells attenuates lung injury and thus protects the lung barrier structure. Dong et al. (51) proved that Nrf2 can inhibit ferroptosis by upregulating the levels of telomerase reverse transcriptase (TERT) and SLC7A11, and then attenuate lung injury induced by intestinal ischemia-reperfusion. Peng et al. (43) revealed that Jumonji domain containing protein-3(JMJD3) deficiency alleviates LPS-induced ALI/ARDS by promoting Nrf2 expression to inhibit the ferroptosis of alveolar epithelial cells. Consistent with previous findings, our experimental results revealed that EA stimulation at the ST36 acupoint significantly inhibited ferroptosis and attenuated LPS-induced ALI/ARDS. Additionally, in LPS-treated MLE-12 cells, we found that LPS stimulation downregulated the expression of GPX4, SLC7A11 and FTH1, increased the levels of MDA and ROS, and reduced the content of GSH. However, the administration of the α7nAchR agonist PNU-282987 inhibited the occurrence of LPS-triggered ferroptosis events. We also found that application of the ferroptosis agonist erastin reversed the inhibitory effect of α7nAchR activation on ferroptosis *in vivo* and *in vitro* experiments, which further demonstrated that EA stimulation exerted a pulmonary protective effect through α7nAchR-mediated inhibition of LPS-induced ferroptosis.

Previous studies have shown that EA treatment can exert anti-inflammatory and antioxidative stress effects, which in turn protect vital organ function (52, 53). However, it is unclear how EA stimulation at the ST36 acupoint remotely modulates the functional state of the organs. Our results showed that EA stimulation at the ST36 acupoint remarkably inhibited LPS-induced ferroptosis events and attenuated the pulmonary inflammatory response. The ST36 acupoint at the hind limb region is mainly located near the sciatic nerve and its branches, and previous studies have demonstrated that the stimulatory signal from EA stimulation may be transmitted into the spinal cord and brain *via* sciatic afferent nerve fibres. Interestingly, our results found that surgical severance of the bilateral sciatic nerve effectively eliminated the inhibitory effect of EA on LPS-triggered ferroptosis events in lung tissues, which was mainly manifested in the downregulation of the expression of GPX4, SLC7A11 and FTH1, the elevation of iron, MDA and ROS levels, and the reduction of reductant GSH content. Additionally, sciatic nerve transection abolished the mitigating effect of EA on the LPS-induced pulmonary inflammatory response. Thus, it was further confirmed that the anti-ferroptosis and pulmonary protective effects of EA treatment in LPS-induced ALI/ARDS act through the sciatic nerve. The vagus nerve is the predominant parasympathetic nerve connecting the brain with most internal organs, and studies have shown that the vagus nerve is the major innervating nerve of the pulmonary airway, acting as a bridge between the central nervous system and the lung (54). To investigate whether the vagus nerve is involved in regulating the transmission of stimulatory signals at the ST36 acupoint into the lungs, our data further confirmed that surgical severance of the left cervical vagus nerve almost completely abolished the inhibitory effect of EA on LPS-induced ferroptosis events and the pulmonary inflammatory response. Therefore, these results suggest that sciatic nerve activity and vagal nerve stimulation induced by EA treatment may share a common set of neural codes to modulate the functional state of the lungs.

In conclusion, our results revealed for the first time that EA stimulation at the ST36 acupoint inhibits LPS-induced ferroptosis of alveolar epithelial cells through activation of α7nAchR, attenuating the pulmonary inflammatory response and thereby alleviating LPS-induced ALI/ARDS.

## Data Availability Statement

The original contributions presented in the study are included in the article/**Supplementary Material**. Further inquiries can be directed to the corresponding authors.

## Ethics Statement

The animal study was reviewed and approved by the Animal Care and Use Committee of the Tongji University School of Medicine.

## Author Contributions

XL, HY, and YZ designed the experiments. YZ, LZ, and HD drafted the manuscript. YZ performed animal experiment, iron, GSH and MDA levels detection, and statistical analysis. LZ, HD, and DF performed western blotting and real-time qPCR. SH, WX, and LZ assisted with the cell experiment. WZ, YW, and KM conducted the assessment of cell viability, ROS detection. QZ, YC, and HZ contributed to critical review and manuscript revision. All authors participated in the article writing and approved the final version of the manuscript.

## Funding

This work was supported by the National Natural Science Foundation of China (No. 82000085, No.81871601 and No.82100090); Shanghai “Rising Stars of Medical Talent” Youth Development Program: Outstanding Youth Medical Talents; the Young Elite Scientist Sponsorship Program by CAST (2018QNRC001); the Basic Research Program for Young Elite Scientist by Shanghai Association for the Study of Pain(2018SASP01); the Research Program for Young Scientist by Shanghai Society of Anesthesiology (2019SSA) and Sponsored by Shanghai Sailing Program (21YF1438400).

## Conflict of Interest

The authors declare that the research was conducted in the absence of any commercial or financial relationships that could be construed as a potential conflict of interest.

## Publisher’s Note

All claims expressed in this article are solely those of the authors and do not necessarily represent those of their affiliated organizations, or those of the publisher, the editors and the reviewers. Any product that may be evaluated in this article, or claim that may be made by its manufacturer, is not guaranteed or endorsed by the publisher.

## References

[1] Dolmatova E Forrester S Wang K Ou Z Williams H Joseph G. Endothelial Poldip2 Regulates Sepsis-Induced Lung Injury *via* Rho Pathway Activation. Cardiovasc Res (2021) cvab295. doi: 10.1093/cvr/cvab295 34528082 PMC9612795

[2] Sevransky J Levy M Marini J. Mechanical Ventilation in Sepsis-Induced Acute Lung Injury/Acute Respiratory Distress Syndrome: An Evidence-Based Review. Crit Care Med (2004) 32:S548–553. doi: 10.1097/01.ccm.0000145947.19077.25 15542963

[3] Ranieri V Rubenfeld G Thompson B Ferguson N Caldwell E Fan E. Acute Respiratory Distress Syndrome: The Berlin Definition. JAMA (2012) 307(23):2526–33. doi: 10.1001/jama.2012.5669 22797452

[4] Fan E Brodie D Slutsky A. Acute Respiratory Distress Syndrome: Advances in Diagnosis and Treatment. JAMA (2018) 319(7):698–710. doi: 10.1001/jama.2017.21907 29466596

[5] Bellani G Laffey J Pham T Fan E Brochard L Esteban A. Epidemiology, Patterns of Care, and Mortality for Patients With Acute Respiratory Distress Syndrome in Intensive Care Units in 50 Countries. JAMA (2016) 315(8):788–800. doi: 10.1001/jama.2016.0291 26903337

[6] Longhurst J. Defining Meridians: A Modern Basis of Understanding. J Basic Clin Physiol Pharmacol (2010) 3(2):67–74. doi: 10.1016/s2005-2901(10)60014-3 20633518

[7] Liu S Wang Z Su Y Ray R Jing X Wang Y. Somatotopic Organization and Intensity Dependence in Driving Distinct NPY-Expressing Sympathetic Pathways by Electroacupuncture. Neuron (2020) 108(3):436–50.e437. doi: 10.1016/j.neuron.2020.07.015 32791039 PMC7666081

[8] Yang N Yang J Ye Y Huang J Wang L Wang Y. Electroacupuncture Ameliorates Intestinal Inflammation by Activating α7nachr-Mediated JAK2/STAT3 Signaling Pathway in Postoperative Ileus. Theranostics (2021) 11(9):4078–89. doi: 10.7150/thno.52574 PMC7977469 33754049

[9] Huang D Chen M Wang Z Hou L Yu W. Electroacupuncture Pretreatment Attenuates Inflammatory Lung Injury After Cardiopulmonary Bypass by Suppressing NLRP3 Inflammasome Activation in Rats. Inflammation (2019) 42(3):895–903. doi: 10.1007/s10753-018-0944-y 30680695

[10] Zou Y Bhat O Yuan X Li G Huang D Guo Y. Release and Actions of Inflammatory Exosomes in Pulmonary Emphysema: Potential Therapeutic Target of Acupuncture. J Inflamm Res (2021) 14:3501–21. doi: 10.2147/jir.S312385 PMC8318722 34335040

[11] Andersson U. The Cholinergic Anti-Inflammatory Pathway Alleviates Acute Lung Injury. Mol Med (2020) 26(1):64. doi: 10.1186/s10020-020-00184-0 32600316 PMC7322708

[12] Chavan S Pavlov V Tracey K. Mechanisms and Therapeutic Relevance of Neuro-Immune Communication. Immunity (2017) 46(6):927–42. doi: 10.1016/j.immuni.2017.06.008 PMC5578398 28636960

[13] Pereira M Leite P. The Involvement of Parasympathetic and Sympathetic Nerve in the Inflammatory Reflex. J Cell Physiol (2016) 231(9):1862–9. doi: 10.1002/jcp.25307 26754950

[14] Yang Z Yin Q Olatunji O Li Y Pan S Wang D. Activation of Cholinergic Anti-Inflammatory Pathway Involved in Therapeutic Actions of α-Mangostin on Lipopolysaccharide-Induced Acute Lung Injury in Rats. Int J Immunopathol Pharmacol (2020) 34:2058738420954941. doi: 10.1177/2058738420954941 32886564 PMC7485160

[15] Engel O Akyüz L da Costa Goncalves A Winek K Dames C Thielke M. Cholinergic Pathway Suppresses Pulmonary Innate Immunity Facilitating Pneumonia After Stroke. Stroke (2015) 46(11):3232–40. doi: 10.1161/strokeaha.115.008989 26451017

[16] Maouche K Polette M Jolly T Medjber K Cloëz-Tayarani I Changeux J. {Alpha}7 Nicotinic Acetylcholine Receptor Regulates Airway Epithelium Differentiation by Controlling Basal Cell Proliferation. Am J Pathol (2009) 175(5):1868–82. doi: 10.2353/ajpath.2009.090212 PMC2774052 19808646

[17] Ding N Wei Q Deng W Sun X Zhang J Gao W. αelectroacupuncture Alleviates Inflammation of Dry Eye Diseases by Regulating the 7nachr/NF-B Signaling Pathway. Oxid Med Cell Longev (2021) 2021:6673610. doi: 10.1155/2021/6673610 33897942 PMC8052151

[18] Jiang T Wu M Zhang Z Yan C Ma Z He S. Electroacupuncture Attenuated Cerebral Ischemic Injury and Neuroinflammation Through α7nachr-Mediated Inhibition of NLRP3 Inflammasome in Stroke Rats. Mol Med (2019) 25(1):22. doi: 10.1186/s10020-019-0091-4 31117961 PMC6530013

[19] Zhang L Wu Z Zhou J Lu S Wang C Xia Y. Electroacupuncture Ameliorates Acute Pancreatitis: A Role for the Vagus Nerve-Mediated Cholinergic Anti-Inflammatory Pathway. Front Mol Biosci (2021) 8:647647. doi: 10.3389/fmolb.2021.647647 34055878 PMC8155617

[20] Wang Z Hou L Yang H Ge J Wang S Tian W. Electroacupuncture Pretreatment Attenuates Acute Lung Injury Through α7 Nicotinic Acetylcholine Receptor-Mediated Inhibition of HMGB1 Release in Rats After Cardiopulmonary Bypass. Shock (Augusta Ga.) (2018) 50(3):351–9. doi: 10.1097/shk.0000000000001050 PMC6072368 29117064

[21] Gahring L Myers E Dunn D Weiss R Rogers S. Nicotinic Alpha 7 Receptor Expression and Modulation of the Lung Epithelial Response to Lipopolysaccharide. PLoS One (2017) 12(4):e0175367. doi: 10.1371/journal.pone.0175367 28384302 PMC5383308

[22] Bayır H Anthonymuthu T Tyurina Y Patel S Amoscato A Lamade A. Achieving Life Through Death: Redox Biology of Lipid Peroxidation in Ferroptosis. Cell Chem Biol (2020) 27(4):387–408. doi: 10.1016/j.chembiol.2020.03.014 32275865 PMC7218794

[23] Wang Y Zhao Y Wang H Zhang C Wang M Yang Y. Histone Demethylase KDM3B Protects Against Ferroptosis by Upregulating SLC7A11. FEBS Open bio (2020) 10(4):637–43. doi: 10.1002/2211-5463.12823 PMC7137800 32107878

[24] Liu G Xu X Tao S Gao M Hou Z. HBx Facilitates Ferroptosis in Acute Liver Failure *via* EZH2 Mediated SLC7A11 Suppression. J Biomed Sci (2021) 28(1):67. doi: 10.1186/s12929-021-00762-2 34615538 PMC8495979

[25] Kajarabille N Latunde-Dada G. Programmed Cell-Death by Ferroptosis: Antioxidants as Mitigators. Int J Mol Sci (2019) 20(19):4968. doi: 10.3390/ijms20194968 PMC6801403 31597407

[26] Xu B Wang H Chen Z. Puerarin Inhibits Ferroptosis and Inflammation of Lung Injury Caused by Sepsis in LPS Induced Lung Epithelial Cells. Front Pediatr (2021) 9:706327. doi: 10.3389/fped.2021.706327 34422728 PMC8371381

[27] Tan C Gurien S Royster W Aziz M Wang P. Extracellular CIRP Induces Inflammation in Alveolar Type II Cells *via* TREM-1. Front Cell Dev Biol (2020) 8:579157. doi: 10.3389/fcell.2020.579157 32984356 PMC7484489

[28] Guohua F Tieyuan Z Xinping M Juan X. Melatonin Protects Against PM2.5-Induced Lung Injury by Inhibiting Ferroptosis of Lung Epithelial Cells in a Nrf2-Dependent Manner. Ecotoxicol Environ Saf (2021) 223:112588. doi: 10.1016/j.ecoenv.2021.112588 34364124

[29] Xu Y Li X Cheng Y Yang M Wang R. Inhibition of ACSL4 Attenuates Ferroptotic Damage After Pulmonary Ischemia-Reperfusion. FASEB J (2020) 34(12):16262–75. doi: 10.1096/fj.202001758R 33070393

[30] Li G Li X Dong J Han Y. Electroacupuncture Ameliorates Cerebral Ischemic Injury by Inhibiting Ferroptosis. Front Neurol (2021) 12:619043. doi: 10.3389/fneur.2021.619043 33763013 PMC7982901

[31] Torres-Rosas R Yehia G Peña G Mishra P del Rocio Thompson-Bonilla M Moreno-Eutimio M. Dopamine Mediates Vagal Modulation of the Immune System by Electroacupuncture. Nat Med (2014) 20(3):291–5. doi: 10.1038/nm.3479 PMC3949155 24562381

[32] Zhai Q Lai D Cui P Zhou R Chen Q Hou J. Selective Activation of Basal Forebrain Cholinergic Neurons Attenuates Polymicrobial Sepsis-Induced Inflammation *via* the Cholinergic Anti-Inflammatory Pathway. Crit Care Med (2017) 45(10):e1075–82. doi: 10.1097/ccm.0000000000002646 PMC5598911 28806219

[33] Kim T Kim S Lee S. Stimulation of the α7 Nicotinic Acetylcholine Receptor Protects Against Sepsis by Inhibiting Toll-Like Receptor *via* Phosphoinositide 3-Kinase Activation. J Infect Dis (2014) 209(10):1668–77. doi: 10.1093/infdis/jit669 24298024

[34] Shibata Y Yasui H Higashikawa K Miyamoto N Kuge Y. Erastin, a Ferroptosis-Inducing Agent, Sensitized Cancer Cells to X-Ray Irradiation *via* Glutathione Starvation *In Vitro* and *In Vivo* . PLoS One (2019) 14(12):e0225931. doi: 10.1371/journal.pone.0225931 31800616 PMC6892486

[35] Deng H Wu L Liu M Zhu L Chen Y Zhou H. Bone Marrow Mesenchymal Stem Cell-Derived Exosomes Attenuate LPS-Induced ARDS by Modulating Macrophage Polarization Through Inhibiting Glycolysis in Macrophages. Shock (Augusta Ga.) (2020) 54(6):828–43. doi: 10.1097/shk.0000000000001549 32433208

[36] Shimouchi A Yokota H Ono S Matsumoto C Tamai T Takumi H. Neuroprotective Effect of Water-Dispersible Hesperetin in Retinal Ischemia Reperfusion Injury. Jpn J Ophthalmol (2016) 60(1):51–61. doi: 10.1007/s10384-015-0415-z 26407617 PMC4713330

[37] Vida G Peña G Deitch E Ulloa L. α7-Cholinergic Receptor Mediates Vagal Induction of Splenic Norepinephrine. J Immunol (2011) 186(7):4340–6. doi: 10.4049/jimmunol.1003722 PMC3083451 21339364

[38] Shen H Meng Y Liu D Qin Z Huang H Pan L. α7 Nicotinic Acetylcholine Receptor Agonist PNU-282987 Ameliorates Cognitive Impairment Induced by Chronic Intermittent Hypoxia. Nat Sci Sleep (2021) 13:579–90. doi: 10.2147/nss.S296701 PMC8123952 34007230

[39] Lin W Jia D Fu C Zheng Y Lin Z. Electro-Acupuncture on ST36 and SP6 Acupoints Ameliorates Lung Injury Via Sciatic Nerve in a Rat Model of Limb Ischemia-Reperfusion. J Inflamm Res (2020) 13:465–70. doi: 10.2147/jir.S264093 PMC7455772 32904499

[40] Bhattacharya J Matthay M. Regulation and Repair of the Alveolar-Capillary Barrier in Acute Lung Injury. Annu Rev Physiol (2013) 75:593–615. doi: 10.1146/annurev-physiol-030212-183756 23398155

[41] Li H Yao C Shi K Zhao Y Du J Hu D. Astragaloside IV Attenuates Hypoxia/Reoxygenation Injury-Induced Apoptosis of Type II Alveolar Epithelial Cells Through miR-21-5p. Bioengineered (2021) 12(1):7747–54. doi: 10.1080/21655979.2021.1982845 PMC8806943 34617873

[42] Xu Z Huang Y Zhou J Deng X He W Liu X. Current Status of Cell-Based Therapies for COVID-19: Evidence From Mesenchymal Stromal Cells in Sepsis and ARDS. Front Immunol (2021) 12:738697. doi: 10.3389/fimmu.2021.738697 34659231 PMC8517471

[43] Peng J Fan B Bao C Jing C. JMJD3 Deficiency Alleviates Lipopolysaccharide−Induced Acute Lung Injury by Inhibiting Alveolar Epithelial Ferroptosis in a Nrf2−dependent Manner. Mol Med Rep (2021) 24(5):1–8. doi: 10.3892/mmr.2021.12447 34542160

[44] Su X Lee J Matthay Z Mednick G Uchida T Fang X. Activation of the Alpha7 Nachr Reduces Acid-Induced Acute Lung Injury in Mice and Rats. Am J Respir Cell Mol Biol (2007) 37(2):186–92. doi: 10.1165/rcmb.2006-0240OC PMC1976545 17431097

[45] Wang J Yao L Wu X Guo Q Sun S Li J. Protection Against Doxorubicin-Induced Cardiotoxicity Through Modulating iNOS/ARG 2 Balance by Electroacupuncture at PC6. Oxid Med Cell Longev (2021) 2021:6628957. doi: 10.1155/2021/6628957 33824696 PMC8007344

[46] Shi Y Dai Q Ji B Huang L Zhuang X Mo Y. Electroacupuncture Pretreatment Prevents Cognitive Impairment Induced by Cerebral Ischemia-Reperfusion *via* Adenosine A1 Receptors in Rats. Front Aging Neurosci (2021) 13:680706. doi: 10.3389/fnagi.2021.680706 34413765 PMC8369428

[47] Wei L Su Y Tan S Zou Y Tang Y Kong G. Electroacupuncture Stimulation at Yanglingquan Acupoint Ameliorates Hepatic Ischemia-Reperfusion Injury by Down-Regulating ET-1 to Inhibit TAK1-JNK/p38 Pathway. Am J Physiol Gastrointest Liver Physiol (2021) 321(6):G690. doi: 10.1152/ajpgi.00012.2021 34788160

[48] Wang Y Xue M Xia Y Jiang Q Huang Z Huang C. Electroacupuncture Treatment Upregulates α7nachr and Inhibits JAK2/STAT3 in Dorsal Root Ganglion of Rat With Spared Nerve Injury. J Pain Res (2019) 12:1947–55. doi: 10.2147/jpr.S203867 PMC6613452 31308727

[49] Wang Z Liu T Yin C Li Y Gao F Yu L. Electroacupuncture Pretreatment Ameliorates Anesthesia and Surgery-Induced Cognitive Dysfunction via Activation of an α7-Nachr Signal in Aged Rats. Neuropsychiatr Dis Treat (2021) 17:2599–611. doi: 10.2147/ndt.S322047 PMC8370114 34413646

[50] Li S Zheng L Zhang J Liu X Wu Z. Inhibition of Ferroptosis by Up-Regulating Nrf2 Delayed the Progression of Diabetic Nephropathy. Free Radical Biol Med (2021) 162:435–49. doi: 10.1016/j.freeradbiomed.2020.10.323 33152439

[51] Dong H Xia Y Jin S Xue C Wang Y Hu R. Nrf2 Attenuates Ferroptosis-Mediated IIR-ALI by Modulating TERT and SLC7A11. Cell Death Dis (2021) 12(11):1027. doi: 10.1038/s41419-021-04307-1 34716298 PMC8556385

[52] Tian L Song S Zhu B Liu S. Electroacupuncture at ST-36 Protects Interstitial Cells of Cajal *via* Sustaining Heme Oxygenase-1 Positive M2 Macrophages in the Stomach of Diabetic Mice. Oxid Med Cell Longev (2018) 2018:3987134. doi: 10.1155/2018/3987134 29854081 PMC5944261

[53] Cai M Lee J Yang E. Electroacupuncture Attenuates Cognition Impairment *via* Anti-Neuroinflammation in an Alzheimer's Disease Animal Model. J Neuroinflamm (2019) 16(1):264. doi: 10.1186/s12974-019-1665-3 PMC6909515 31836020

[54] Kavoussi B Ross B. The Neuroimmune Basis of Anti-Inflammatory Acupuncture. Integr Cancer Ther (2007) 6(3):251–7. doi: 10.1177/1534735407305892 17761638

