# Supplementary material for: Electroacupuncture Alleviates LPS-Induced ARDS Through α7 Nicotinic Acetylcholine Receptor-Mediated Inhibition of Ferroptosis
Source: Front Immunol. 2022 Feb 10;13:832432. doi: 10.3389/fimmu.2022.832432 (PMC8866566; doi:10.3389/fimmu.2022.832432)
Supplement: Supplementary file 1 [file DataSheet_1.docx]

| Gene Primer Sequence (5’-3’) |
| --- |
| β-actin Forward AGTGTGACGTTGACATCCGT  Reverse GCAGCTCAGTAACAGTCCGC |
| IL-1β Forward GCAACTGTTCCTGAACTCAACT  Reverse ATCTTTTGG GGTCCGTCAACT |
| TNF-α Forward AAGCCTGTAGCCCACGTCGTA  Reverse GGCACCACTAGTTGGTTGTCTTTG |
| Gpx4 Forward TTCTCAGCCAAGGACATCGA  Reverse AGGCCAGGATTCGTAAACCA |
| SLC7A11 Forward TTGGAGCCCTGTCCTATGC  Reverse CGAGCAGTTCCACCCAGAC |
| FTH1 Forward CCATCAACCGCCAGATCAAC  Reverse GCCACATCATCTCGGTCAAA |

**Supplementary table 1** Real-time qPCR primer sequences


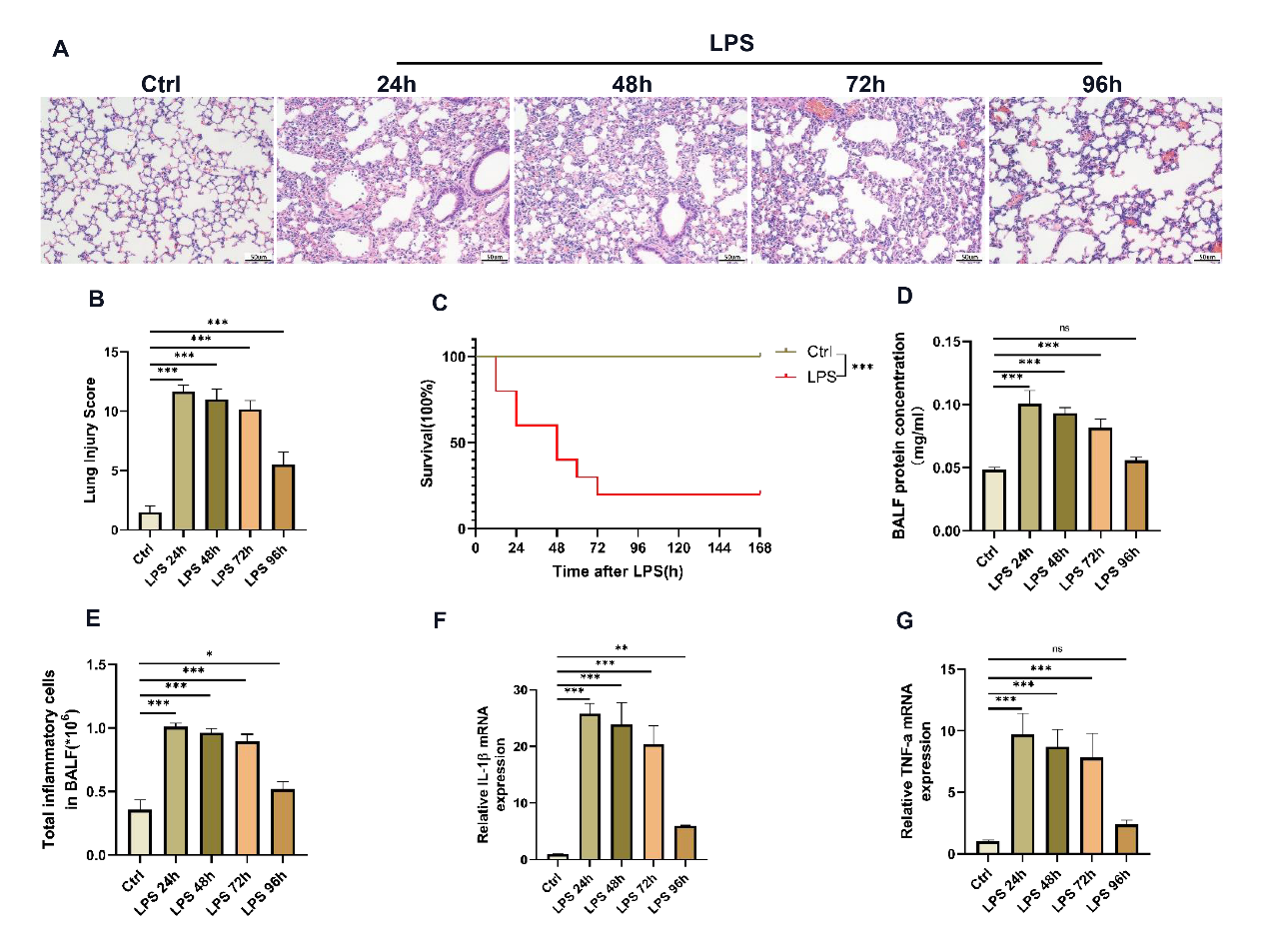


**Supplementary Fig. 1** The changes of lung injury at different time points in the LPS-induced ALI/ARDS mouse model. **A** The representative H&E staining of lung tissue sections (scale bar, 50 μm). **B** The lung injury score analysis (n=6). **C** The survival rate of mice (n=10). **D** The protein concentration in BALF (n=6). **E** The number of inflammatory cells in BALF (n=6). The IL-1β (**F**) and TNF-α (**G**) mRNA levels in lung tissues were determined by real-time qPCR (n=6).
